# Refractive Index Sensing-Based Sensitivity Enhancement Using Surface Plasmon Resonance Sensor with Integration of Tin Diselenide and Zirconium Diselenide

**DOI:** 10.3390/s26134279

**Published:** 2026-07-05

**Authors:** Rajeev Kumar, Pushkar Praveen, Biswajit Brahma, Paolo Barsocchi, Akash Kumar Bhoi

**Affiliations:** 1 Department of Electronics and Communication, Engineering, Graphic Era (Deemed to Be University), Dehradun 248001, Uttarakhand, India; rajeevkrc@gmail.com; 2 Department of Electronics & Communication Engineering, G. B. Pant Institute of Engineering and Technology, Pauri 246194, Uttarakhand, India; 3 McKesson Corporation, 32559 Lake Bridgeport St., Fremont, CA 94555, USA; biswajit.brahma@gmail.com; 4 Institute of Information Science and Technologies, National Research Council, 56124 Pisa, Italy; 5 Symbiosis Institute of Technology, Pune Campus, Symbiosis International (Deemed to Be University), Pune 412115, Maharashtra, India; akashkrbhoi@gmail.com

**Keywords:** surface plasmon resonance, Tin Diselenide, Zirconium Diselenide, sensitivity, detection accuracy

## Abstract

**Highlights:**

- Proposed a highly sensitive multilayer SPR sensor using Ag/SnSe_2_/ZrSe_2_ structure with Kretschmann configuration.
- The maximum sensitivities of 337.98°/RIU and 320.94°/RIU were achieved at analyte RI of 1.34 and 1.35, respectively.
- The integration of SnSe_2_ and ZrSe_2_ layers significantly enhanced the electromagnetic field confinement and plasmonic interactions.
- The optimized Ag and SnSe_2_ thicknesses improved the minimum reflectance, detection accuracy, and figure of merit.
- The numerical FEM and TMM analyses confirmed the stronger evanescent field penetration and enhanced sensing performance.
- The proposed sensor shows strong potential for biomedical diagnostics, chemical sensing, and environmental monitoring applications.

**Abstract:**

A highly sensitive surface plasmon resonance (SPR) sensor is theoretically presented, including Silver (Ag), Tin Diselenide (SnSe_2_), Zirconium Diselenide (ZrSe_2_) and a sensing layer using the Kretschmann configuration. At the optimized thickness of the Ag layer, the sensitivity was measured using the angular interrogation method with a refractive index (RI) of 1.33–1.35. The sensitivity of the sensor was found to be 337.98°/RIU for a 2 nm SnSe_2_ layer thickness at RI of 1.34 and 320.94°/RIU for a 1 nm SnSe_2_ layer thickness at RI of 1.35 throughout with remarkable figure of merit (FoM) of 60.78/RIU and 64.57/RIU at 633 nm wavelength. The maximum sensitivity was achieved with 1 nm thickness of the SnSe_2_ layer. By systematically optimizing the Ag thickness, significant improvements in sensitivity, minimum reflectance (R_min_), detection accuracy (DA), and figure of merit (FoM) were achieved compared with the conventional Ag-only configuration. These additional SnSe_2_ layers increase the confinement of the electromagnetic field, increase the number of adsorption sites for biomolecules, and increase the effective change in the RI, resulting in larger shifts in the resonance angles. The proposed multilayer sensor provides a promising platform for high-performance, stable, and repeatable biosensing applications in chemical detection, environmental monitoring, and medical diagnostics, according to the results obtained.

## 1. Introduction

The application of surface plasmon resonance (SPR) in industrial, chemical, environmental, and biological sciences has led to its widespread use in recent decades [1,2]. However, several methods have been used for sensing, including interferometry [3], grating couplers, spectroscopic methods [4], and waveguide methods [5]. However, these methods are time-consuming, challenging to set up, and somewhat demanding. SPR is a more dependable and approachable method for overcoming these challenges. The excitation of surface plasmons (SPs) results in a resonance condition called SPR [6]. Additionally, the oscillation of the electron charge density at the metal–dielectric interface excites SPs, which are transverse magnetic waves. The resonance condition is reached when the propagation constants of the SP and evanescent waves are equal. Although this resonance condition causes a sharp dip in the reflected or transmitted light, direct incident light cannot excite SPs because the wave vector of the incident light is smaller than that of the SPs. Various geometries have been employed to enhance the wave vector of the incident light [7]. The most popular geometry is the Kretschmann configuration [8]. To improve the wave vector of the incident light and excite SPs, Kretschmann introduced a modified Otto configuration [9] in 1971. A layer of plasmonic metal and a dielectric medium were applied to the prism base. When the incident energy is transferred to the SPs at a particular angle, the SPs are excited, and the reflected light is collected at the opposite face of the prism. The incident light hits one face of the high-index prism. The resonance angle is the precise angle at which the least amount of reflected energy is received. The resonance angle depends on the additional layers and dielectric properties of the sensing medium. Consequently, the minimum reflectance for each dielectric medium was observed at a distinct resonance angle. SPR’s unique features are utilized in sensing applications. Furthermore, SPR is a key platform for biological and chemical molecule interactions that benefit the environment. There are additional justifications for applying the SPR technique, such as high stability, accurate detection, affordability, ease of use, and label-free status. SPR sensors have a variety of uses, and Liedberg et al. [10] first discussed the use of SPR sensors in various fields in 1983. Gupta et al. [11] discussed a number of additional fiber-based SPR sensor applications. They have utilized the phenomenon whereby changes in the analyte concentration or RI cause changes in the resonance angle. Several parameters were examined. The sensitivity of the sensor is directly proportional to the resonance angle shift, which is sensitive to changes in the SM RI. In conventional SPR, the generation of SPs and adsorption of biomolecules or other analytes are accomplished solely by a thin layer of noble metal. The preferred metal, gold, exhibits stable adsorption of analytes with high sensitivity and is free from oxidation, corrosion, and chemical instability. However, the DA decreases because of a wider resonance curve [12]. Ag exhibits a sharper reflectance curve and higher DA, but its chemical stability is poor because it oxidizes rapidly when directly exposed to the atmosphere. Ag can be effectively used in SPR sensors if a protective layer is applied to prevent oxidation. Conventional SPR sensors that use a prism, metal layer, and sensing medium provide lower sensitivity. Therefore, by functionalizing a metal layer with a two-dimensional (2D) layered nanomaterial, its sensitivity can be increased to detect smaller biomolecules and other analytes. Additionally, current research indicates that the use of 2D layered nanomaterials has increased exponentially. Graphene [13], transition metal dichalcogenides (TMDs) [14], and black phosphorus (BP) [15]-based SPR sensors have been used to identify microbes, biomolecules, and various analytes. The optical, electrical, and electronic properties of 2D layered nanomaterials are particularly intriguing, making them suitable for ultrahigh-sensitivity analyte sensing. They are layered because of their precise stacking on the metal coating. Their special advantages, such as a higher surface area, higher density of charge carriers, and higher adsorption energies, allow for more accurate and ultrasensitive analyte detection than traditional SPR sensors. Due to the limited interaction between the evanescent field and the SM, conventional Ag-based SPR sensors typically have moderate sensitivity. Depending on the operating wavelength and structural arrangement, conventional SPR configurations typically yield sensitivity values between 60 and 200°/RIU. Several researchers have used 2D materials like graphene, MoS_2_, WS_2_, black phosphorus, and transition metal dichalcogenides in SPR structures to improve electromagnetic field confinement and analyte interaction in order to get around these restrictions [13,14,15]. The effectiveness of 2D-material-assisted SPR sensing has been demonstrated by recent studies that have reported significantly improved sensitivities using hybrid multilayer structures.

Recently, to improve the sensing performance and stability, SPR sensors frequently use the SnSe_2_ layer. SnSe_2_ is a 2D semiconductor that is a member of the TMD family [16]. It has a large surface area, strong light–matter interaction, and high RI. In an SPR structure, the introduction of a thin SnSe_2_ layer above the metal layer increases the interaction between the evanescent plasmonic field and SM by improving the confinement of the electromagnetic field at the metal–dielectric interface. When the RI of the analyte varies, this results in increased sensitivity and a more pronounced change in the resonance angle. Furthermore, the SnSe_2_ layered structure offers a large number of active sites for biomolecule adsorption, increasing the effectiveness of analyte binding. Additionally, the SnSe_2_ layer can serve as a protective layer that reduces metal oxidation and enhances the chemical stability of the sensor. SnSe_2_ can be readily incorporated into multilayer SPR configurations to enhance the sensitivity, DA, and overall sensor performance because of its ultrathin thickness and tunable optical properties.

The layered TMDC ZrSe_2_ is distinguished by its unique optical and electrical properties [17]. Similar to other dichalcogenides, this substance crystallizes in a hexagonal structure and is known for its semiconducting properties. An intriguing feature of ZrSe_2_ for use in electronic and optoelectronic devices is its indirect bandgap. In the field of 2D material science, the capacity to exfoliate ZrSe_2_ into thin layers or monolayers generates opportunities and may lead to advancements in flexible and transparent electronic devices. ZrSe_2_ is noteworthy for its mechanical and thermal stabilities, in addition to its electrical properties. The development of optical components, such as waveguides and photodetectors, where the exact control of light–matter interactions is critical, depends on the investigation of their optical properties, especially the RI.

The suggested SPR sensor offers a platform for possible biosensing applications and is mainly intended for RI sensing. Nevertheless, biomolecular interactions, binding kinetics, and analyte-specific selectivity are not specifically modeled in this study. Depending on the target analyte, the ZrSe_2_ surface can be functionalized using suitable receptor molecules, such as antibodies, enzymes, aptamers, or DNA probes, for useful biosensing applications. For instance, DNA probes can be used to detect nucleic acids, enzyme-based functionalization can enable glucose sensing, and antibody-functionalized surfaces can be used to detect cancer biomarkers. ZrSe_2_ is a good platform for this kind of functionalization because of its large surface area and advantageous adsorption properties. Through enhanced electromagnetic confinement and analyte adsorption capability, the ZrSe_2_ layer also significantly contributes to the improvement of plasmonic interaction and sensing performance, even though the optimization study mainly focuses on the thickness of the SnSe_2_ layer. The combined effect of SnSe_2_ and ZrSe_2_ layers, where SnSe_2_ improves field confinement and ZrSe_2_ contributes to stronger light–matter interaction and effective analyte coupling, results in the enhanced sensing performance of the suggested structure.

## 2. Theory of Sensor Structure, Fabrication Process, Refractive Index, Modeling and Performance Matrices

### 2.1. Theory of Sensor Structure and Fabrication Process

**Figure 1:**
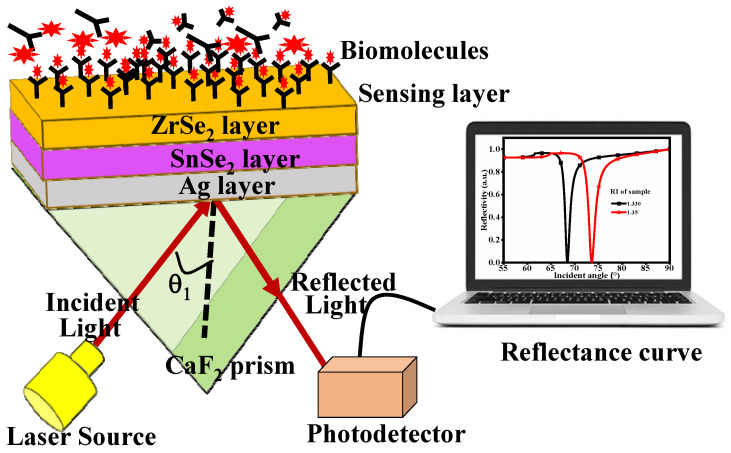
Sensor Structure.

A prism-coupled SPR biosensor using the Kretschmann configuration is proposed. At a particular incidence angle, a p-polarized laser beam was incident on the CaF_2_ prism and struck the thin Ag layer, as shown in Figure 1. SPs are collective oscillations that are excited when the momentum of the incident photons and free electrons at the Ag surface match. This resulted in a sharp decrease in the reflected intensity, represented by a dip in the reflectance curve. Phase matching and effective light coupling to the plasmon mode were made possible by the CaF_2_ prism. The depth and sharpness of the resonance were significantly influenced by the thickness of the Ag layer, which served as the main plasmon-supporting Ag metal. Some 2D materials like SnSe_2_ and ZrSe_2_ have been added on top of Ag to improve performance. ZrSe_2_ increases the light–matter interaction close to the sensing interface and offers a large active surface for immobilizing biomolecules, while the SnSe_2_ layer enhances electromagnetic field confinement and fosters a stronger interaction between the analyte and the evanescent wave. The RI changes when biomolecules attach to the top surface, altering the resonance angle and plasmon propagation constants. Highly sensitive detection is made possible by the photodetector, which tracks this shift and shows it as a movement of the dip in the reflectance curve. Therefore, compared to a conventional single-metal SPR structure, the addition of extra monolayers results in stronger adsorption, larger resonance shifts, better signal quality, and lower detection limits.

**Figure 2:**
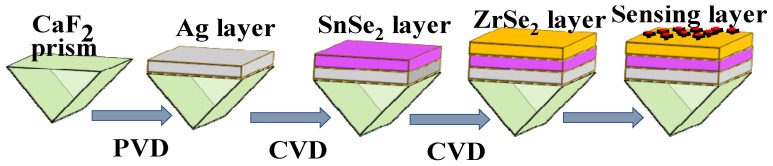
Fabrication process.

The detailed fabrication process of the SPR sensor based on the CaF_2_ prism is shown in Figure 2. Prism preparation and cleaning are the first steps in the process, which are essential because any contamination or surface roughness can interfere with plasmon excitation and DA. To remove organic residues, the prism is usually cleaned in stages using acetone, isopropyl alcohol, deionized water, and piranha solutions [18]. This is followed by nitrogen drying. To increase the surface energy and encourage strong adhesion of the deposited layers, UV–ozone or oxygen plasma treatment is sometimes employed. A clear and smooth prism ensures stable optical coupling, low scattering loss, and good repeatability between devices. In the absence of support, the Ag layer may eventually peel, crack, or drift, resulting in unstable resonance angles and poor repeatability. This is addressed by introducing an extremely thin layer of titanium (Ti) or chromium (Cr), usually ranging from 1–3 nm, between the prism and Ag. The adhesion material, typically introduced by PVD techniques such as thermal evaporation or sputtering [19], is deposited first after the prism has been thoroughly cleaned (acetone → isopropyl alcohol → DI water → nitrogen drying, occasionally plasma/UV-ozone treatment). Similar to glue, Cr and Ti create robust chemical bonds with the Ag layer and prism surface. This incredibly thin layer enhances the mechanical robustness without appreciably interfering with plasmon excitation. However, if it is excessively thick, it can dampen the SPs and increase optical absorption, generating a shallower and wider reflectance dip. Therefore, thickness optimization is essential for achieving high performance. Following cleaning, Physical Vapor Deposition (PVD) was used to deposit a thin layer of Ag. PVD generates a homogeneous, precisely regulated nanometer-thick layer by vaporizing Ag metal in a high-vacuum chamber and condensing it onto the prism surface. Because Ag thickness significantly affects the SPR performance, such as sensitivity, precise thickness control is important. Long-term stability is enhanced by PVD’s dense layers, superior adhesion, and high reproducibility of PVD. The next step involves growing the SnSe_2_ layer, usually by Chemical Vapor Deposition (CVD) [20,21]. In CVD, a continuous thin layer grows by the reaction or breakdown of precursor gases on a heated substrate. This technique allows for good control over stoichiometry, crystallinity, and large-area uniformity. The sensitivity was increased by the SnSe_2_ layer enhancement of the electromagnetic field confinement and the provision of active sites for analyte interaction. Similarly, ZrSe_2_ is deposited on top of SnSe_2_, usually by CVD [22,23]. This layer enhances the light–matter interaction close to the sensing interface, increases the surface area, and encourages biomolecule immobilization. To prevent flaws or excessive thickness that could dampen plasmon waves, the sequential growth of 2D materials must be carefully optimized. Finally, the top surface of the ZrSe_2_ layer served as the sensing interface, where biomolecule attachment occurred. ZrSe_2_ is a two-dimensional layered material that can immobilize biomolecules such as proteins, antibodies, DNA strands, and hormones because of its high adsorption capacity and large surface area. Biomolecules attach to the functionalized ZrSe_2_ surface through chemical bonding, electrostatic interactions, or physical adsorption when the sensor surface is exposed to a biological sample. The RI changes near the metal–dielectric interface as a result of this attachment, creating a thin layer of analyte above the ZrSe_2_ layer. A discernible shift in the SPR resonance angle resulted from the change in RI, which altered the resonance condition of the SPs generated at the Ag metal layer. The adsorption of even a small concentration of analytes results in a significant resonance shift because ZrSe_2_ increases the electromagnetic field and offers strong interactions with biomolecules. Consequently, in the SPR sensor configuration, the ZrSe_2_ layer serves as an efficient biorecognition and sensing platform, allowing for extremely sensitive biomolecule detection. It is crucial to remember that the fabrication procedure outlined in this work is conceptual and meant to show that the suggested SPR sensor is practically feasible. The current study is based on numerical simulations and is entirely theoretical. In order to reduce optical damping and complexity, adhesion layers such as Ti or Cr—often used in experimental fabrication to increase the adhesion of the metal to the prismatic substrate—are excluded from the simulation model. They will be considering in future experimental investigations and may slightly modify resonance properties. The simulation assumes an ideal, smooth and homogeneous interface between the layers. Practical factors such as surface roughness, grain boundaries and interfacial dispersion are omitted in order to simplify and focus on the basic plasmonic behaviour of the proposed structure. The performance of the sensor may be affected by actual manufacturing conditions such as material insularity, adhesion layer effects and defects in the thin layer.

### 2.2. Thickness Selection, Refractive Index, and Modeling

To determine the ideal condition that offers maximum plasmonic coupling and superior sensing performance, the Ag thickness was varied between 25 and 60 nm. During this time, the Ag layer changes from a comparatively thin, weakly supporting layer to one that can support SP oscillations that are both strong and stable. The most efficient transfer of optical energy from the incident light to the plasmon mode occurs as the thickness approaches the ideal value, resulting in a large resonance angle shift and an exceptionally low R_min_ value. While thicker layers introduce higher absorption losses and limit evanescent field penetration into the analyte, thinner Ag layers result in poor coupling because of incomplete electron confinement. Consequently, the thickness that provides the maximum sensitivity along with a deep and sharp resonance dip was determined by investigating the 25–60 nm range. The RI of Ag is determined by the Drude model as given in Equation (1) below:

(1) $$n_{Ag}={\left(\varepsilon_{r}+\varepsilon_{i}\right)}^{\frac{1}{2}}={\left(1-\frac{\lambda^{2}\lambda_{c}}{\lambda_{p}^{2}(\lambda_{c}+i\lambda )}\right)}^{\frac{1}{2}}$$

where $\lambda_{c}(Collision\;wavelength)$ = 1.7614 × 10^−5^ m and $\lambda_{p}(Plasma\;wavelength)$ = 1.4541 × 10^−7^ m.

The 0–2 nm range was chosen for the thickness of SnSe_2_ in the proposed sensor because it captures the change from a bare Ag metal surface to a few-layer 2D coating, where optical damping is still limited, but plasmon enhancement is strong. An ultrathin SnSe_2_ layer enhances the overlap between the evanescent field and the SM, enhances the surface adsorption sites, and dramatically alters the electromagnetic boundary at the metal–analyte interface. Owing to the improved field confinement and stronger light–matter interaction, the resonance angle shift typically increases as the thickness increases from 0 to 2 nm. Beyond this range, higher absorption and scattering losses, which dampen plasmon oscillations, increase the R_min_ value and widen the reflectance curve. The RI of the SnSe_2_ layer was 3.5018 + 0.28348i [24]. The next layer was a 2D ZrSe_2_ material. Regarding the thickness of ZrSe_2_ in the proposed SPR sensor, the proposed SPR sensor’s ZrSe_2_ layer thickness of 0.253 nm was chosen because it roughly matches the monolayer thickness of this 2D material. Because they maintain a strong coupling between the incident light and SP waves generated at the metal–dielectric interface, ultrathin 2D materials are typically preferred for SPR-based sensing structures. The interaction between the plasmonic field and analyte molecules adhered to the sensing surface is enhanced by the monolayer ZrSe_2_ layer, which permits the evanescent electromagnetic field to efficiently enter the SM. The thickening of the ZrSe_2_ layer may result in additional optical absorption and scattering losses that broaden the resonance curve and dampen SP waves, ultimately decreasing the sensitivity and DA of the sensor. Consequently, the sensing performance of the SPR sensor was enhanced by choosing an ultrathin thickness of 0.253 nm, which ensured strong electromagnetic field confinement, minimal plasmon damping, and effective biomolecule interactions. The RI of the ZrSe_2_ layer was 3.4890 + 1.7705i [25]. The RI range of 1.33–1.35 is frequently chosen in SPR biosensing because it reflects aqueous biological media and the minute changes that occur when biomolecules attach to the sensor surface. When proteins, nucleic acids, lipids, or other solutes are present, the effective RI slightly increases based on the concentration and molecular weight. At optical wavelengths, pure water is near 1.33. Therefore, disease detection using SPR usually involves measuring minute increments caused by specific binding events rather than a large RI. For instance, in immunosensing, the local RI may move from approximately 1.33 to 1.34 or higher because of antibody attachment and subsequent antigen capture from blood or serum. Measurable RI increases during this period can be caused by elevated protein content linked to infectious disease markers, cancer biomarkers (such as PSA, CA-125, and HER2), or inflammatory disorders. The mass density close to the metal surface is also changed by DNA hybridization, virus attachment, or bacterial adhesion, which pushes the refractive index back to 1.35. Changes in this limited window can result from changes in plasma composition or glucose levels.

A theoretical model of the 2D multilayer structure of the prism-based SPR sensor is presented in this study. This model focuses on the total internal reflection phenomenon. The best possible structure is required for the sensor design. The layer thickness was simulated and optimized using the N-layer model. The N-layer model structure is shown in Figure 1. As illustrated in Figure 1, all layers were stacked along the Z-direction in accordance with the N-layer model. A relationship exists between the tangential fields at the first and last boundaries. Equations (2)–(8) were used to calculate the reflectivity.

(2) U1V1=MUN−1VN−1

where the components of the electric and magnetic fields first and last are denoted by U_1_, V1, U_N-1,_ and V_N-1_ respectively. The characteristic matrix of the combined structure is denoted by M, as shown in Equation (3).

(3) $$M=\prod_{k=2}^{N-1}M_{k}=\left[\begin{array}{cc} M_{11} & M_{12}\\ M_{21} & M_{22} \end{array}\right]$$

Here, M_k_ is written as shown in Equation (4):

(4) $$M_{k}=\left[\begin{array}{cc} \cos\beta_{k} & -i\sin\beta_{k}/q_{k}\\ -iq_{k}\sin\beta_{k} & \cos\beta_{k} \end{array}\right]$$

where β_k_ = optical constant, q_k_ = phase thickness, λ = wavelength (633 nm) and θ_1_ = angle of incident, given in Equations (5) and (6):

(5) $$q_{k}=\sqrt{\frac{\mu_{k}}{\varepsilon_{k}}}\cos\theta_{k}=\frac{\sqrt{\varepsilon_{k}-n_{1}^{2}\sin^{2}\theta_{1}}}{\varepsilon_{k}}$$

(6) $$\beta_{k}=\frac{2\pi }{\lambda }n_{k}\cos\theta_{k}\left(z_{k}-z_{k-1}\right)=\frac{2\pi d_{k}}{\lambda }\sqrt{\varepsilon_{k}-n_{1}^{2}\sin^{2}\theta_{1}}$$

The reflection coefficient (r_p_) formula is shown in Equation (6):

(7) $$r_{p}=\frac{\left(M_{11}+M_{12}q_{n}\right)q_{1}-\left(M_{21}+M_{22}q_{n}\right)}{\left(M_{11}+M_{12}q_{n}\right)q_{1}+\left(M_{21}+M_{22}q_{n}\right)}$$

The complex dielectric constant of the k-th layer is represented by ε_k_ in the above Equation (6). It is defined as ε_k_ = (n_k_ + ik_k_)^2^, where n_k_ and k_k_ are the RI and extinction coefficient, respectively. Each layer’s wave vector component is represented by q_k_, and the propagation constant along the interface is represented by β_k_. The free-space wave number is represented by k_0_, and the incident angle is represented by θ_1_. The layer number is indicated by the subscript k, where k = 1, 2, 3, … denotes prism, metal, 2D material, and SM, respectively.

The reflection coefficient for p-polarized light is calculated using the TMM as given in Equation (7). The reflectance is obtained as Equation (8), where r_p_ represents the Fresnel reflection coefficient.

(8) $$R={\left|r_{p}\right|}^{2}$$

### 2.3. Performances Matrices

The above equations describe the distribution of the electric and magnetic fields in a multilayer SPR structure using TMM. Equation (9) represents the electric field E_y_ and magnetic field H_y_ components in the first layer (usually the prism), where the fields are expressed in terms of the incident magnetic field $H_{y}^{inc}$ and the reflection coefficient r_p_ for p-polarized light [26].

(9) $$\left[\begin{array}{c} H_{y1}(z)\\ E_{y1}(z) \end{array}\right]=P_{1}\left(z\right)\left[\begin{array}{c} 1+r_{p}\\ q_{1}(1-r_{p}) \end{array}\right]H_{y}^{inc},z_{1}\leq z\leq z_{2}$$

Matrix P_1_(z) is the propagation matrix, as given in Equation (10), which describes how electromagnetic waves propagate through the first layer. It contains trigonometric terms $cos(\beta_{k\;at\;z})$ and $sin(\beta_{k\;at\;z})$ that represent phase variation along the thickness of the layer, while $q_{1}$ is related to the wave impedance of that layer.

$P_{1}\left(z\right)\;is$ written as:

(10) $$P_{1}\left(z\right)=\left[\begin{array}{cc} cos(\beta_{k\;at\;z}) & \left(i/q_{1})sin(\beta_{k\;at\;z}\right)\\ iq_{1}sin(\beta_{k\;at\;z}) & cos(\beta_{k\;at\;z}) \end{array}\right]$$

Equation (11) extends this formulation to subsequent layers j ≥ 2j (such as metals, 2D materials, and sensing media).

(11) $$\left[\begin{array}{c} H_{yj}(z)\\ {-E}_{yj}(z) \end{array}\right]=P_{j}\left(z=z_{j}+d_{j}\right)\left[\begin{array}{c} 1+r_{p}\\ q_{1}(1-r_{p}) \end{array}\right]H_{y}^{inc},z_{j}\leq z\leq z_{j+1}$$

The matrix Pj (z) (Equation (12) represents the propagation of the electromagnetic wave inside layer j, where d_j_ is the thickness of that layer and $q_{j}$ is the corresponding wave impedance parameter. These matrices can be used to calculate the electric and magnetic field distributions across the multilayer structure, which aids in determining the resonance condition, reflectance, and sensing performance of the SPR sensor.

(12) $$P_{j}\left(z\right)=\left[\begin{array}{cc} cos(\beta_{k(at\;z=z-z_{j})}) & \left(i/q_{j})sin(\beta_{k(at\;z=z-z_{j})}\right)\\ iq_{j}sin(\beta_{k(at\;z=z-z_{j})}) & cos(\beta_{k(at\;z=z-z_{j})}) \end{array}\right]$$

The performance parameters of the sensor must be measured to design an improved sensor. Thus, the performance parameters of the SPR sensor, including sensitivity, FWHM, DA, FOM, and PD, were calculated. It is defined as the resonance angle change (Δθ_res_) per unit change in the analyte’s RI (Δn_s_) and is mathematically represented as shown in Equation (13).

(13) $$S=\frac{\Delta \theta_{Res.}}{\Delta n_{s}}\;\left({}^{\circ}/RIU\right)$$

The DA of the SPR sensor was another parameter used in this study. This shows the amount of information that a sensor can detect precisely and accurately. This is the mathematical definition of DA, as shown in Equation (14).

(14) $$DA=\frac{1}{FWHM}(1/{}^{\circ})$$

The FOM is calculated as the product of the sensitivity and the DA, as expressed in Equation (15):

(15) $$FoM=S\times DA\;\left(/RIU\right)$$

## 3. Result Analysis

### 3.1. Measurement of the Field Analysis

**Figure 3:**
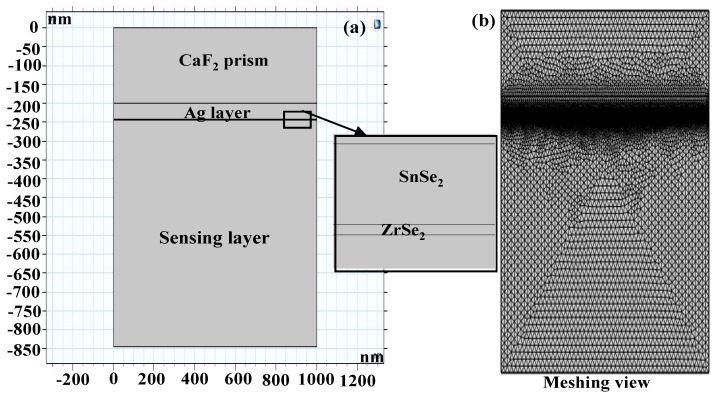
(**a**) Schematic diagram of the proposed structure. (**b**) Finite element meshing view of the simulation domain.

To precisely assess the electromagnetic field distribution, a numerical analysis of the proposed SPR sensor was performed using the finite element method (FEM) in COMSOL Multiphysics 6.1. A multilayer structure with prism, Ag layer, SnSe_2_, ZrSe_2_, and SM arranged in the Kretschmann configuration makes up the simulation domain. With lateral dimensions spanning multiple wavelengths of the incident light, the domain size was selected to be sufficiently large to prevent boundary-induced artifacts. To ensure precise numerical computation, the proposed structure uses a nonuniform triangular mesh analysis. To minimize the computational complexity, a relatively coarser mesh was employed in the prism and sensing regions, whereas a finer mesh was applied close to the metal and 2D material interfaces, where the surface plasmon waves were strongly confined. To precisely capture the rapidly changing electromagnetic fields, a nonuniform triangular mesh was used, with incredibly fine mesh elements applied close to the metal/2D material interfaces. In order to minimize computational costs, triangular meshing was employed away from the interface, while the minimum mesh element size close to the interface was set to the sub-nanometer scale. For plasmon excitation, the propagation of p-polarized incident light from the CaF_2_ prism toward the metal layer was modeled using appropriate electromagnetic boundary conditions. The incident electromagnetic wave was introduced at the input boundary using a port or scattering boundary condition, and the lateral boundaries were given scattering conditions to reduce undesired reflections. The continuity of the tangential electric and magnetic field components is automatically satisfied at each interface between the layers to ensure proper wave propagation through the multilayer system. In addition, a Perfectly Matched Layer was applied above the sensing region to remove artificial reflections from the computational boundary and absorb outgoing electromagnetic waves. By acting as an absorbing domain and attenuating the evanescent fields that extend into the SM, the PML makes it possible to accurately simulate the open-domain SPR sensing environment and ensure a reliable sensor performance evaluation. In order to eliminate artificial reflections and absorb outgoing waves, PMLs were applied at the simulation domain’s outer boundaries. To ensure effective absorption, the PML thickness was chosen to be roughly one wavelength of the incident light. At the proper domain edges, scattering boundary conditions were implemented. Figure 3a shows the schematic configuration of the proposed SPR sensor structure. The structure consists of a high-RI CaF_2_ prism at the top, which is used to couple the incident light to the plasmonic metal layer through the Kretschmann configuration. Beneath the prism, a thin Ag layer was deposited, which acted as the plasmonic material responsible for exciting SPPs at the metal–dielectric interface. Below the Ag layer, two ultrathin 2D materials, SnSe_2_, and ZrSe_2_ were introduced before the SM. These 2D layers enhance the interaction between the electromagnetic field and analyte molecules owing to their high surface area and strong charge transfer capability. The sensing layer represents the analyte region, whose RI changes during detection. The inset highlights the stacked arrangement of the SnSe_2_ and ZrSe_2_ layers near the Ag layer, showing their position at the metal–SM interface, where SPP excitation occurs. The meshing view used in the numerical simulation is shown in Figure 3b. To precisely solve Maxwell’s electromagnetic equations, a fine triangular mesh was created throughout the computational domain. The electromagnetic field varies rapidly in these areas owing to SPP excitation, which makes the mesh much denser near the metal and 2D material interfaces. However, areas farther from the interface, where the field variation is smoother, employ comparatively larger mesh elements. This adaptive meshing technique ensures accurate computation of the electric field distribution, resonance behavior, and sensing performance of the SPR structure while increasing the numerical accuracy and convergence of the simulation while preserving computational efficiency.

**Figure 4:**
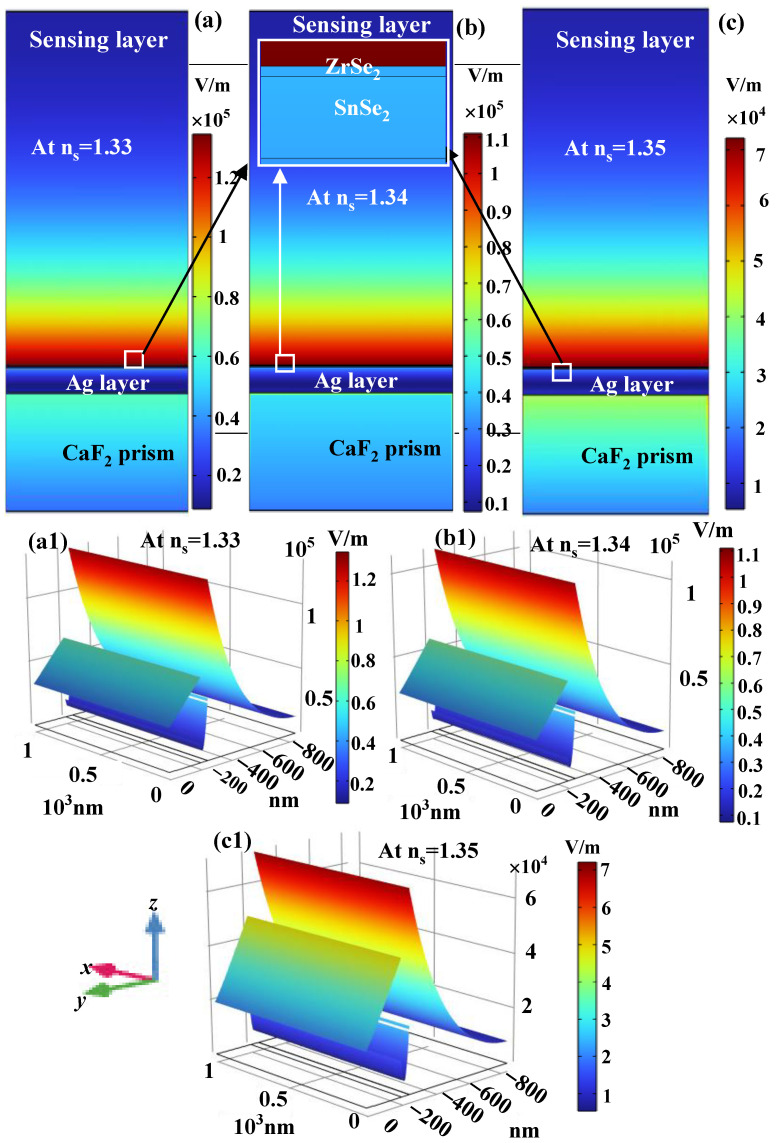
Electric field distribution 2D plot (**a**) at ns = 1.33, (**b**) at n_s_ = 1.34, and (**c**) at n_s_ = 1.35; 3D plot (**a1**) at n_s_ = 1.33, (**b1**) at n_s_ = 1.34, and (**c1**) at n_s_ = 1.35.

The electric field distribution in the proposed SPR sensor with a CaF_2_ prism, Ag layer, SnSe_2_ layer, and SM for various analyte RIs (n_s_ = 1.33, 1.34, and 1.35) is depicted in Figure 4a–c. The electric field intensity is represented by a color scale, with red denoting a strong field enhancement and blue denoting a weak field intensity. The electric field is strongly confined close to the Ag–SnSe_2_ interface in Figure 4a for n_s_ = 1.33, indicating effective surface plasmon excitation. An essential feature of SPR sensing is the penetration of the evanescent field from the metal layer into the SM. Because of the exponential decay of the evanescent field, the high-intensity region (red/yellow) indicates that the plasmonic wave is concentrated at the interface and progressively decays away from the surface into the analyte region. The resonance condition in Figure 4b for n_s_ = 1.34 is altered by a small shift in the refractive index of the sensing layer. This caused a slight shift in the distribution of the electric field and a change in the maximum field intensity at the interface. This suggests that the surface plasmon wave is extremely sensitive to changes in the RI of the SM. The electric field peak is further altered, and the evanescent field PD into the SM increases in Figure 4c for n_s_ = 1.35. This indicates that the resonance angle shift used for detection in SPR sensors is caused by a strong interaction between the analyte and plasmonic field. The spatial variation in the electric field intensity across the multilayer structure is further demonstrated by the 3D plots in Figure 4a1–c1. The maximum electric field enhancement at the metal–dielectric interface and its gradual decay along the propagation direction are clearly visible in these plots. The ability of the sensor to detect minute changes in the RI of the SM is demonstrated by the strong localization of the electromagnetic field at the interface, which validates effective plasmon excitation.

**Figure 5:**
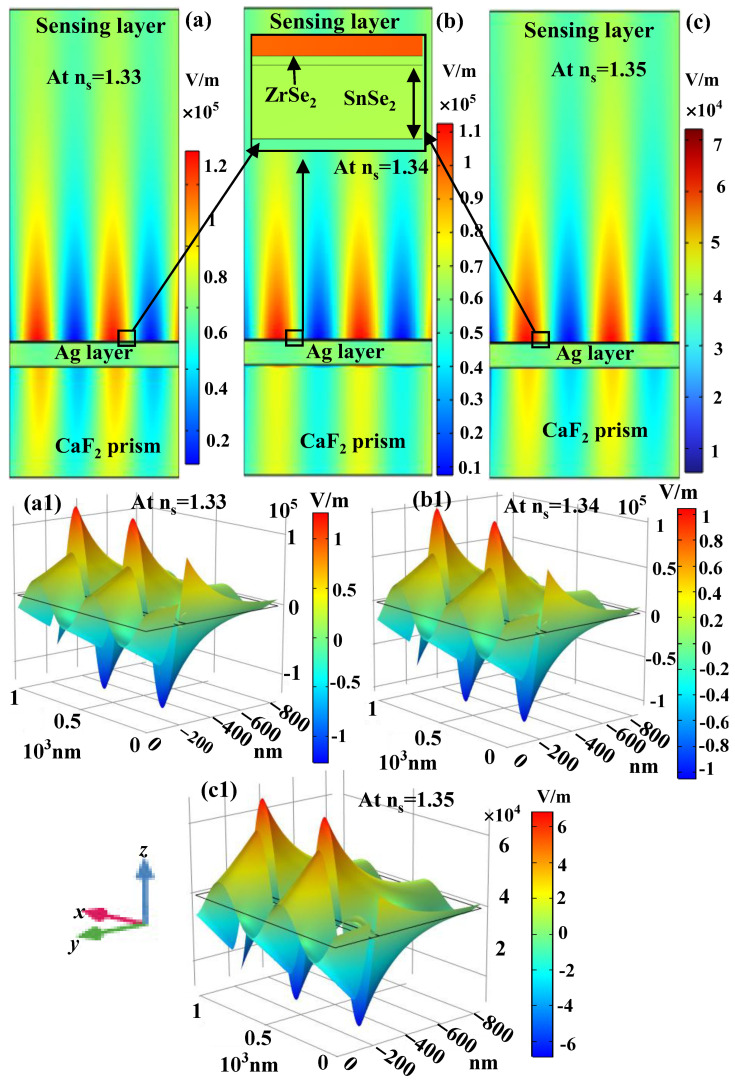
SPP mode 2D plot (**a**) at n_s_ = 1.33, (**b**) at n_s_ = 1.34, and (**c**) at n_s_ = 1.35; 3D plot (**a1**) at n_s_ = 1.33, (**b1**) at n_s_ = 1.34, and (**c1**) at n_s_ = 1.35.

The SPP mode of the suggested sensor structure is shown in Figure 5 for various analyte RIs (n_s_ = 1.33–1.35). The spatial distribution of the electric field intensity throughout the proposed structure is depicted in the 2D field plots in Figure 5a–c, where the color scale denotes the magnitude of the electric field (blue denotes lower field intensity and red denotes strong field enhancement). The Ag–SnSe_2_–ZrSe_2_ interface exhibited strong localization of the maximum electric field, indicating effective excitation of the SPPs. This strong localization occurs because the incident p-polarized light from the CaF_2_ prism couples with the free electrons in the Ag layer to generate SP waves that travel along the metal–dielectric interface. The ZrSe_2_ and SnSe_2_ layers increased the confinement of the evanescent electromagnetic field close to the sensing interface and improved the light–matter interaction. A discernible shift in the electric field distribution was observed as the RI rises from 1.33 to 1.35. A stronger interaction between the plasmonic field and analyte molecules is indicated by an increase in the intensity and penetration depth (PD) of the evanescent field into the sensing region. Because even a slight change in the analyte RI modifies the plasmon resonance condition and changes the field distribution, this behavior validates the high RI sensitivity of the proposed structure. The spatial variation in the SPP mode is further demonstrated by the 3D plots in Figure 5a1–c1, which exhibit a prominent electric field peak at the metal–2D material interface and a fast exponential decay away from the interface along the vertical direction. This distinctive decay shows that the electromagnetic energy is tightly contained close to the sensing interface, which is a basic property of SP waves. The improved field confinement of the proposed structure increases the interaction between the plasmonic field and SM, which directly improves the sensing performance. Thus, the suggested structure provides strong plasmon excitation, high field confinement, and improved analyte interaction, which improves the sensitivity and DA of the SPR sensor, according to the electric field distribution analysis.

**Figure 6:**
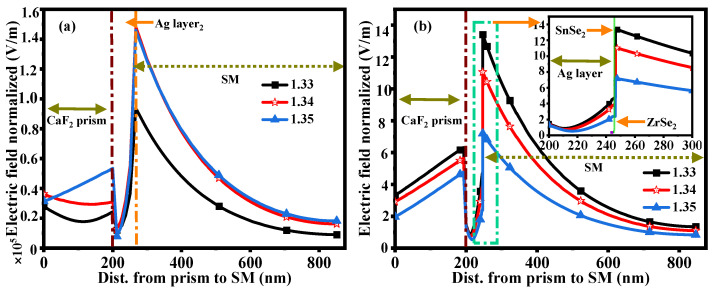
Electric field normalized (**a**) conventional (CaF_2_–Ag–SM) sensor. (**b**) Proposed structure.

The normalized electric field normalized of the Surface Plasmon Polaritons (SPPs) mode for RIs n_s_ = 1.33 and 1.35 is plotted against the distance between the prism and the SM in Figure 6. A clear comparison of the field intensity and penetration behavior in various layers of the structure is made possible by normalizing the electric field with respect to the maximum electric field at the metal–dielectric interface. Figure 6a, which corresponds to the conventional structure (CaF_2_–Ag–SM), shows that the excitation of the SPP mode causes the electric field to exhibit a sharp peak close to the Ag layer interface. Following the evanescent field behavior, the field exponentially decayed into the SM after reaching its maximum value. The 1/e factor, which indicates the distance at which the electric field intensity drops to 1/e (approximately 37%) of its maximum value, can be used to characterize the field’s decay. The effective interaction region between the plasmonic field and analyte is determined by this distance, which is referred to as the PD. The peak normalized electric field shifts and slightly increases as the RI increases from 1.33 to 1.35, suggesting stronger plasmon coupling. The normalized electric field near the interface in Figure 6b, which depicts the suggested structure with extra layers (proposed structure), is noticeably stronger than that in the conventional structure. The field enhancement within the ZrSe_2_ and SnSe_2_ layers, which enhances plasmon excitation and electromagnetic confinement, is shown in the inset. Additionally, a slightly longer 1/e decay length was indicated by the evanescent field extending farther into the SM. The light–matter interaction at the sensing interface is enhanced by this stronger and longer field interaction, which improves the sensing performance and increases the sensitivity of the proposed SPR sensor. The maximum electric field intensity and PD for the conventional and suggested SPR structures at various RI are summarized in Table 1. The peak electric field intensity for the conventional structure increased dramatically with RI, peaking at 148,244.6 V/m at RI = 1.34 with a PD of 215.24 nm. In contrast, the proposed structure exhibited a moderate PD of 167.675 nm and a higher peak intensity at RI = 1.33 (134,305V/m), suggesting stronger field confinement near the sensing interface. The PD in the suggested structure increased to 217.785 nm as the RI increased to 1.35. This improves the sensing performance by strengthening the interaction between the evanescent field and SM. It should be mentioned that better sensing performance is not always ensured by deeper penetration. The degree of electromagnetic field confinement and the intensity of plasmon–analyte interaction close to the sensing interface have a significant impact on the sensitivity of an SPR sensor.

### 3.2. Sensitivity and Minimum Reflectance Performances

**Figure 7:**
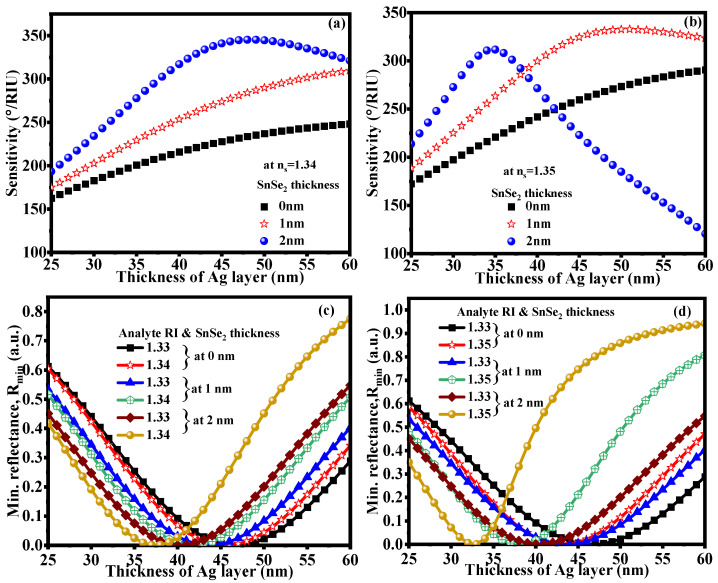
Sensitivity vs. Ag thickness at (**a**) n_s_ = 1.34 and (**b**) n_s_ = 1.35; R_min_ vs. Ag thickness (**c**) n_s_ = 1.33 & 1.34 and (**d**) n_s_ = 1.33 & 1.35 with various SnSe_2_ thicknesses.

For two RIs of the SM, n_s_ = 1.34 and n_s_ = 1.35, Figure 7 illustrates the sensitivity of the SPR sensor with respect to the thickness of the Ag layer while considering varying SnSe_2_ thicknesses (0, 1, and 2 nm). The plots clearly show that the sensitivity is not monotonic but rather reaches an optimal value at a specific Ag thickness, where the coupling between the SPs and incident light reaches its maximum. Considering n_s_ = 1.34, as the Ag thickness increases from lower values, the Ag layer becomes continuous and supports stronger plasmon oscillations, increasing the sensitivity, as shown in Figure 7a. Once the sensitivity reached an optimal region, which was approximately in the mid-thickness range, it began to decline. A thicker Ag layer weakens the ability of the evanescent field to penetrate the analyte region and increases optical damping, which causes this reduction. The sensitivity is still the lowest when the SnSe_2_ layer is absent (0 nm) because there is less interaction with the biomolecules. The response was improved by adding 1 nm SnSe_2_ because of the improved field confinement and increased charge transfer at the interface. The best performance was observed for 2 nm SnSe_2_, where a greater resonance shift was produced by a stronger adsorption capacity and better overlap between the plasmon field and SM. Because n_s_ = 1.35 (Figure 7b), there are some variations in the ideal thickness. A higher surrounding RI shifts the condition for the strongest coupling by altering the plasmon-propagation constant. Consequently, the Ag thickness that offers peak sensitivity undergoes a slight shift. Once again, the performance was significantly improved by adding SnSe_2_. According to the curves, the sensitivity increased with the SnSe_2_ thickness up to the optimal value; thereafter, more material would probably cause losses and widen the resonance. For various SnSe_2_ thicknesses and SM RIs, Figure 7c,d show that R_min_ varies with the Ag film thickness. R_min_ is a measure of the SPR dip’s depth and the effectiveness with which incident light energy is converted to SPs; a lower value denotes a stronger coupling and higher resonance quality. When comparing the curves for n_s_ = 1.33 and 1.34 in Figure 7c, it is evident that R_min_ decreases as the Ag thickness approaches the ideal region in the mid-thickness range. The photon–plasmon momentum matching was at its peak at this point, producing a very deep dip. R_min_ increases again when the Ag layer is thicker because thicker layers increase absorption losses and stop the evanescent field from penetrating into the sensing region, while thinner Ag layers are not continuous enough to support SP oscillations. The impact of SnSe_2_ is clear: as its thickness increases, the dip tends to go deeper, close to the optimum, because the extra layer strengthens the field confinement and enhances the interaction at the metal–dielectric interface. It is crucial to remember that this study’s variation in Ag and SnSe_2_ thickness is meant for parametric optimization and comprehending how structural parameters affect sensing performance. Prior to analyte detection in practical applications, the SPR sensor needs to have a fixed configuration. Nevertheless, additional thickness may introduce damping away from the ideal, which explains the sharp rise in R_min_ at higher Ag thicknesses. A similar behavior is shown in Figure 7d when the RI shifts from n_s_ = 1.33 to 1.35; however, the location and size of the minimum shift are different. At a slightly different Ag thickness, the best coupling occurred because a higher surrounding RI altered the plasmon wave vector. Once again, the appropriate SnSe_2_ incorporation considerably lowers R_min_, confirming the enhanced sensor performance and stronger SP excitation.

### 3.3. Reflectance Curve Performances

**Figure 8:**
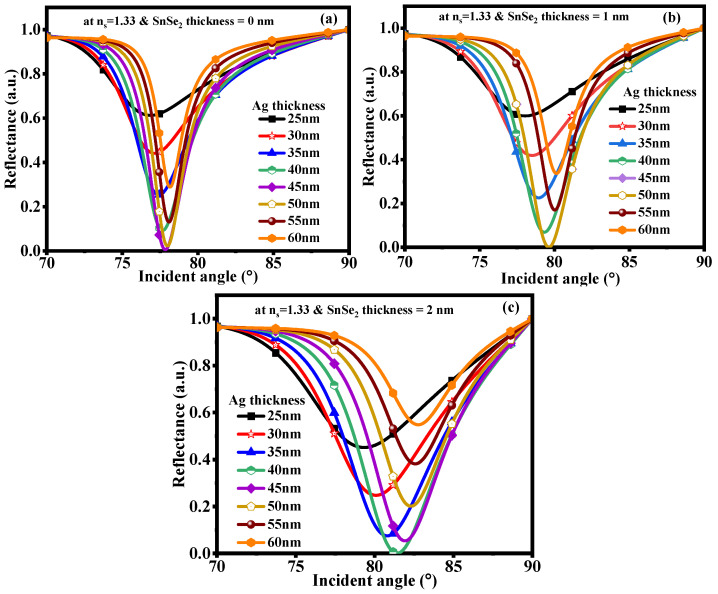
Reflectance curve with n_s_ = 1.330 at various Ag thickness: (**a**) SnSe_2_ thickness = 0 nm, (**b**) SnSe_2_ thickness = 1 nm, and (**c**) SnSe_2_ thickness = 2 nm.

The reflectance curves of the SPR structure are shown in Figure 8 for a sensing medium refractive index of ns = 1.330 at various Ag thicknesses while varying the SnSe_2_ layer. The maximum coupling angle between the incident light and surface plasmons is indicated by the resonance dip in each curve. The plasmon excitation efficiency and sensing performance were significantly influenced by the depth and sharpness of the dip. When SnSe_2_ is not present (0 nm) in Figure 8a, the Ag layer primarily controls the resonance behavior. The dip position and depth fluctuated in response to changes in Ag thickness. Because the metal layer may be partially discontinuous, the coupling is weaker, and the dip is comparatively shallow for thinner layers. Effective energy transfer is indicated by a much deeper and sharper dip as the thickness approaches its ideal value. Once more, the dip becomes wider and less noticeable as the thickness of Ag increases because this causes increased damping and less evanescent field penetration into the analyte. The resonance dips in Figure 8b with 1 nm SnSe_2_ are notably deeper than those in the structure without SnSe_2_. Stronger plasmon excitation is produced by the improved matching conditions of the extra layer and enhancement of the electromagnetic field confinement close to the sensing interface. The modification of the effective RI as perceived by the plasmon wave may also cause a slight shift in the ideal Ag thickness. The enhancement is even more pronounced in the optimal region in Figure 8c for 2 nm SnSe_2_. The coupling was extremely efficient, as evidenced by the minimum reflectance approaching extremely low values. Nevertheless, the losses in the multilayer stack increase, and the dip quality deteriorates once more if the Ag layer becomes overly thick. Therefore, although adding SnSe_2_ enhances the sensitivity and interaction, damping may still be introduced by an excessive overall thickness.

**Figure 9:**
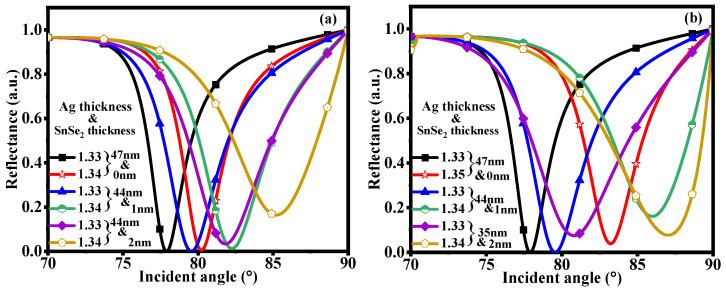
Reflectance curve at R_min_: (**a**) n_s_ = 1.33 & 1.34; (**b**) n_s_ = 1.33 & 1.35.

The reflectance curves assessed under ideal circumstances that produced the lowest reflectance for the two analyte RIs are displayed in Figure 9. These curves provide clear information on how the resonance angle, dip depth, linewidth, and overall sensing quality are affected by the addition of SnSe_2_ and the modification of Ag thickness. The conventional structure without SnSe_2_ (Ag = 47 nm) produced a clear resonance dip with good coupling, as shown in Figure 9a (transition between n_s_ = 1.33 and 1.34). However, the angular shift was relatively small, resulting in a sensitivity of approximately 232.04°/RIU. The sensitivity increases to 270.26°/RIU when 1 nm SnSe_2_ is added, but the dip remains deep, and the shift between RIs increases. A much greater displacement of the resonance angle and a notable increase in sensitivity to 337.98°/RIU were the results of the plasmon field’s even stronger interaction with the SM at 2 nm SnSe_2_. The improvement in the shift takes precedence, resulting in the highest FoM in this set, even though R_min_ increases slightly and the curve widens. A similar enhancement trend can be observed in Figure 9b (transition between n_s_ = 1.33 and 1.35); however, the optimum parameters shift because the plasmon wave vector is dependent on the surrounding RI. With a sensitivity of 265.62°/RIU and a comparatively narrow FWHM, the bare Ag layer exhibited strong coupling once more, providing good DA. The shift is further enhanced to 320.94°/RIU by adding 1 nm SnSe_2_, because of the improved light–matter interaction. The sensitivity remains high (311.60°/RIU) when the thickness is increased to 2 nm, but the resonance widens (larger FWHM), lowering the FoM and DA. This illustrates the well-known trade-off: more damping may be introduced, but the angular shift may be increased by a stronger interaction. These findings are supported by the values in Table 2. Because the evanescent field overlaps more with the analyte when the SnSe_2_ thickness increases, the sensitivity increases. Simultaneously, the FWHM typically increases, causing the dip to widen and the DA (inverse of FWHM) to decrease. Therefore, whether the maximum sensitivity or highest FoM is prioritized determines the best performance. For instance, the greatest sensitivity and FoM are provided by the 2 nm case at n_s_ = 1.34, whereas sharper dips may be obtained from thinner layers. After a thorough assessment of sensitivity, FoM, detection accuracy, and minimum reflectance, one optimized structure is chosen for real-world application. Ag is 44 nm thick, SnSe_2_ is 2 nm thick, and ZrSe_2_ is a monolayer in the optimized configuration. Reliable and stable sensing performance is ensured by this configuration, which offers a fair trade-off between increased sensitivity and acceptable spectral properties. The presence of SnSe_2_ and ZrSe_2_ layers causes additional optical damping even though the suggested multilayer structure greatly increases sensitivity. This leads to a wider resonance curve (higher FWHM), which in turn lowers DA and FoM. On the other hand, because of lower optical losses, the traditional Ag-only structure shows sharper resonance dips with higher FoM values. This behavior demonstrates the inherent trade-off in SPR sensor design, wherein decreased spectral sharpness frequently results from increasing sensitivity through stronger plasmon–analyte interaction.

### 3.4. Conventional Sensor

**Figure 10:**
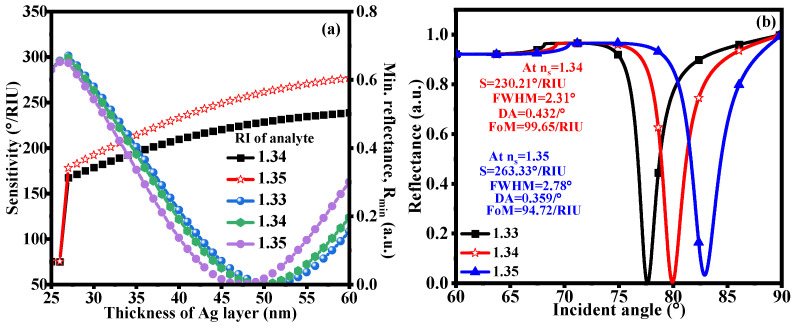
Structure: CaF_2_–Ag–SM (**a**) sensitivity and R_min_ vs. Ag thickness and (**b**) reflectance curve at R_min_ value.

Figure 10 shows how the Ag thickness alone controls the sensing behavior of the conventional single–Ag SPR structure without any additional 2D materials. The sensitivity and R_min_ are plotted as functions of Ag thickness in Figure 10a. Because the Ag layer becomes a continuous support for stronger and more stable SP oscillations, the sensitivity increases rapidly as the thickness increases from lower R_min_ values. Momentum matching between incident photons and plasmons becomes most effective close to the optimal region, which is approximately in the 45–55 nm range. The angular shift for a change in the RI reaches its maximum at this point, and the resonance dip is at its deepest (very small R_min_). Further increasing the thickness of the Ag layer resulted in increased optical absorption within the metal and decreased evanescent field penetration into the analyte. Consequently, the sensitivity decreased, R_min_ increased, and plasmon coupling weakened. This demonstrates the importance of precisely optimizing the Ag thickness, even in simple structures. The reflectance curves for the two RIs corresponding to the optimized R_min_ condition are shown in Figure 10b. The result was a deep and sharp dip, confirming that the surface plasmons were strongly excited. The dip shifts in angle as the refractive index varies, and the sensitivity is calculated using this displacement. Compared to multilayer or 2D-material-assisted designs, the field interaction with the analyte is reduced because only the Ag layer is present; however, the resonance is still clean and narrow, which contributes to good DA. Although the FoM values of the conventional Ag-only SPR sensor are higher, its sensitivity is much lower than that of the suggested structure. Larger resonance angle shifts result from the multilayer configuration’s enhancement of the evanescent field interaction with the analyte. Therefore, the conventional structure might be preferred when higher FoM and sharper resonance properties are needed, while the proposed sensor is better suited for applications requiring high sensitivity.

A comparison of the proposed SPR sensor with previously published research is presented in Table 3. In the RI range of 1.33–1.35, the proposed structure achieves a higher sensitivity of 337.98°/RIU, which is much higher than the values reported in previous studies. The enhanced sensitivity suggests improved plasmonic interaction and better sensing performance of the suggested design, even though the DA (0.179/°) and FoM (60.78/RIU) are comparable to those of previous works. This work provides a more thorough evaluation of sensor performance by taking into account sensitivity, DA, FoM, and physical interaction mechanisms, in contrast to traditional comparisons that only concentrate on sensitivity. On the other hand, by enhancing evanescent field confinement and boosting interaction with the SM, the suggested structure greatly increases sensitivity.

## 4. Conclusions

We present a multi-layer SPR sensor using Ag, SnSe_2_, and ZrSe_2_ layers. The numerical analysis shows that, compared to the conventional Ag-only structure, the addition of SnSe_2_ and ZrSe_2_ layers significantly enhances the interaction between the evanescent field and SM, resulting in larger resonance angle shifts. The sensing capabilities of the sensor fall within the analyte’s 1.33–1.35 RI detection range. This SPR sensor response is linear in the detection range, and the sensitivity is 337.98°/RIU and 320.94°/RIU for RI of 1.34 and 1.35, respectively. Moreover, the maximum PD is 206.04 nm and 217.785 nm at RI of 1.34 and 1.35, respectively. The optimized structure maintained acceptable dip sharpness and stability while significantly increasing the sensitivity and FoM. The findings show that the suggested SPR sensor provides a platform with increased sensitivity and a moderate compromise in FoM and DA. This trade-off is inherent to multilayer plasmonic systems and should be taken into account depending on the intended use, especially in biosensing situations where obtaining incredibly narrow resonance curves is less important than detecting minute changes in RI. Overall, the proposed sensor offers a viable path toward extremely sensitive, dependable, and repeatable biosensing platforms appropriate for chemical analysis, environmental monitoring, and medical diagnostics.

## Figures and Tables

**Table 1:** Measured the maximum electric field and PD.

RI	Conventional Structure		Proposed Structure	
	Peak Intensity (V/m)	PD (nm)	Peak Intensity (V/m)	PD (nm)
1.33	93,782.51	203.02	134,305	167.675
1.34	148,244.6	215.24	110,815	206.045
1.35	145,997.2	213.05	72,025	217.785

**Table 2:** Measuring the performance at RI of 1.34 and 1.35.

RI of Analyte	Ag Thick. (nm)	SnSe_2_ Thick. (nm)	S (°/RIU)	R_min_ at n_s_ = 1.33	R_min_ at n_s_ = 1.34 & 1.35	FWHM (°) n_s_ = 1.34 & 1.35	DA (1/°) n_s_ = 1.34 & 1.35	FoM (/RIU) n_s_ = 1.34 & 1.35
1.34	47	0	232.04	2.08 × 10^−4^	5 × 10^−3^	4.91	0.203	47.26
	44	1	270.26	2.7 × 10^−4^	1.2 × 10^−2^	4.89	0.204	55.26
	44	2	337.98	3.3 × 10^−2^	0.164024	5.56	0.179	60.78
1.35	47	0	265.62	2.08 × 10^−4^	3.9 × 10^−2^	3.91	0.255	67.93
	44	1	320.94	2.7 × 10^−4^	0.161235	4.97	0.201	64.57
	35	2	311.60	7.4 × 10^−2^	7.6 × 10^−2^	6.15	0.162	50.66

**Table 3:** Comparative analysis of proposed and existing work.

Authors and Ref.	RI of Analyte	S (°/RIU)	DA (/°)	FoM (/RIU)
Ansari et al. [27]	1.33–1.35	294.44	-	45.93
Karki et al. [28]	1.33–1.35	295.9	0.238	70.62
Kumar et al. [29]	1.33–1.35	273.05	0.204	-
Pillai et al. [30]	1.33–1.35	235.01	-	-
Proposed work	1.33–1.34 and 1.35	337.98	0.179	60.78

## Data Availability

The dataset generated is already presented in the manuscript and will be made available on request.

## References

[1] Li B., Zhang F., Liu W., Chen X., Gao Y., Wang F., Zhang X., Yan X., Cheng T. (2022). An Ultraviolet Sensor Based on Surface Plasmon Resonance in No-Core Optical Fiber Deposited by Ag and ZnO Film. Surf. Interfaces.

[2] Homola J., Piliarik M. (2006). Surface Plasmon Resonance (SPR) Sensors. Surface Plasmon Resonance Based Sensors.

[3] Homola J. (2003). Present and Future of Surface Plasmon Resonance Biosensors. Anal. Bioanal. Chem..

[4] Pelton J.T., McLean L.R. (2000). Spectroscopic Methods for Analysis of Protein Secondary Structure. Anal. Biochem..

[5] Srivastava T., Jha R., Das R. (2011). High-Performance Bimetallic SPR Sensor Based on Periodic-Multilayer-Waveguides. IEEE Photonics Technol. Lett..

[6] Nylander C., Liedberg B., Lind T. (1982). Gas Detection by Means of Surface Plasmon Resonance. Sens. Actuators.

[7] Kumar R., Pal S., Prajapati Y.K., Kumar S., Saini J.P. (2022). Sensitivity Improvement of a MXene- Immobilized SPR Sensor with Ga-Doped-ZnO for Biomolecules Detection. IEEE Sens. J..

[8] Kretschmann E., Raether H. (1968). Radiative Decay of Non Radiative Surface Plasmons Excited by Light. Z. Naturforsch.-Sect. A J. Phys. Sci..

[9] Otto A. (1968). Excitation of Nonradiative Surface Plasma Waves in Silver by the Method of Frustrated Total Reflection. Z. Phys..

[10] Liedberg B., Nylander C., Lunström I. (1983). Surface Plasmon Resonance for Gas Detection and Biosensing. Sens. Actuators.

[11] Tabassum R., Gupta B.D. (2015). Surface Plasmon Resonance Based Fiber Optic Detection of Chlorine Utilizing Polyvinylpyrolidone Supported Zinc Oxide Thin Films. Analyst.

[12] Wu L., You Q., Shan Y., Gan S., Zhao Y., Dai X., Xiang Y. (2018). Few-Layer Ti3C2Tx MXene: A Promising Surface Plasmon Resonance Biosensing Material to Enhance the Sensitivity. Sens. Actuators B Chem..

[13] Bahmani E., Kaatuzian H., Shafagh S.G. (2026). High-Performance Au–MoS_2_–Graphene Multilayer SPR Biosensor with Superior Sensitivity and Precision. Sci. Rep..

[14] Dey B., Rahman M.T., Saha A. (2026). Enhanced Urine Glucose Sensing Using Two-Dimensional TMDCs-Based SPR Biosensor. Sci. Rep..

[15] Cai Y., Zhang J., Zhou Y., Chen C., Lin F., Wang L. (2021). Refractive Index Sensor with Alternative High Performance Using Black Phosphorus in the All-Dielectric Configuration. Opt. Express.

[16] Singh P., Aggrawal V., Badhulika S. (2024). Synergistic Integration of Ni-Metal Organic Framework/SnS_2_ Nanocomposite and Nickel Foam Electrode for Ultrasensitive and Selective Electrochemical Detection of Albumin in Simulated Human Blood Serum. Nanotechnology.

[17] Rakkini A.P.V., Mohanraj K. (2018). Effect of Different Combinations of Precursors of Zirconium and Selenium in the Electrodeposited ZrSe2 Thin Films. Ionics.

[18] Koh K.S., Chin J., Chia J., Chiang C.L. (2012). Quantitative Studies on PDMS-PDMS Interface Bonding with Piranha Solution and Its Swelling Effect. Micromachines.

[19] Lazauskas A. (2014). Surface Morphology, Cohesive and Adhesive Properties of Physical Vapor Deposited Chromium and Cromium Composite Thin Films. Ph.D. Thesis.

[20] Choi H., Lee J., Shin S., Lee J., Lee S., Park H., Kwon S., Lee N., Bang M., Lee S.-B. (2018). Fabrication of High Crystalline SnS and SnS2 Thin Films, and Their Switching Device Characteristics. Nanotechnology.

[21] Feng J., Chen J., Geng B., Feng H., Li H., Yan D., Zhuo R., Cheng S., Wu Z., Yan P. (2011). Two-Dimensional Hexagonal SnS_2_ Nanoflakes: Fabrication, Characterization, and Growth Mechanism. Appl. Phys. A.

[22] Tian Y., Zheng M., Cheng Y., Yin Z., Jiang J., Wang G., Chen J., Li X., Qi J., Zhang X. (2021). Epitaxial Growth of ZrSe 2 Nanosheets on Sapphire via Chemical Vapor Deposition for Optoelectronic Application. J. Mater. Chem. C.

[23] Mleczko M.J., Zhang C., Lee H.R., Kuo H.-H., Magyari-Köpe B., Moore R.G., Shen Z.-X., Fisher I.R., Nishi Y., Pop E. (2017). HfSe2 and ZrSe2: Two-Dimensional Semiconductors with Native High-κ Oxides. Sci. Adv..

[24] Ermolaev G.A., Yakubovsky D.I., El-Sayed M.A., Tatmyshevskiy M.K., Mazitov A.B., Popkova A.A., Antropov I.M., Bessonov V.O., Slavich A.S., Tselikov G.I. (2021). Broadband Optical Constants and Nonlinear Properties of SnS2 and SnSe2. Nanomaterials.

[25] Zotev P.G., Wang Y., Andres-Penares D., Severs-Millard T., Randerson S., Hu X., Sortino L., Louca C., Brotons-Gisbert M., Huq T. (2023). Van Der Waals Materials for Applications in Nanophotonics. Laser Photon. Rev..

[26] Shalabney A., Abdulhalim I. (2010). Electromagnetic Fields Distribution in Multilayer Thin Film Structures and the Origin of Sensitivity Enhancement in Surface Plasmon Resonance Sensors. Sens. Actuators A Phys..

[27] Ansari G., Kanjariya P., Reddy M.S., Choudhury S., Albert H.M., Kaur I., Rathi V., Sead F.F., Sharma Y., Sinha A. (2025). Refractive Index Sensing-Based Surface Plasmon Resonance Sensor for Sensitivity Enhancement: Theoretical Analysis. Plasmonics.

[28] Karki B., Alsubaie A.S., Kumar R., Reddy M.S., M J.R., Palo H.K., Kumar S. (2026). Enhanced Surface Plasmon Resonance Sensing Using MXene and Tantalum Disulfide-Based Multilayer Structures. Surf. Interfaces.

[29] Kumar R., Agarwal S., Pal N., Pal S., Prajapati Y.K. (2025). Platinum Diselenide (PtSe_2_) Mediated Heterostructure Based SPR Sensor for the Detection of Formalin: A Theoretical Analysis. Phys. Scr..

[30] Pillai A.M., Nair N., Das M.K., Ram S.K. (2025). Strategic Approaches to Enhance Efficiency and Commercial Feasibility of Copper-Based Surface Plasmon Resonance Sensing. Next Mater..

